# A Mechanically-Adaptive Polymer Nanocomposite-Based Intracortical Probe and Package for Chronic Neural Recording

**DOI:** 10.3390/mi9110583

**Published:** 2018-11-08

**Authors:** Allison Hess-Dunning, Dustin J. Tyler

**Affiliations:** 1 Rehabilitation Research and Development, Louis Stokes Cleveland VA Medical Center, Cleveland, OH 44106, USA; 2 Advanced Platform Technology Center, Cleveland, OH 44106, USA; 3 Department of Biomedical Engineering, Case Western Reserve University, Cleveland, OH 44106, USA; dustin.tyler@case.edu

**Keywords:** neural probe, intracortical, microelectrodes, bio-inspired, polymer nanocomposite, cellulose nanocrystals, photolithography, Parylene C

## Abstract

Mechanical, materials, and biological causes of intracortical probe failure have hampered their utility in basic science and clinical applications. By anticipating causes of failure, we can design a system that will prevent the known causes of failure. The neural probe design was centered around a bio-inspired, mechanically-softening polymer nanocomposite. The polymer nanocomposite was functionalized with recording microelectrodes using a microfabrication process designed for chemical and thermal process compatibility. A custom package based upon a ribbon cable, printed circuit board, and a 3D-printed housing was designed to enable connection to external electronics. Probes were implanted into the primary motor cortex of Sprague-Dawley rats for 16 weeks, during which regular recording and electrochemical impedance spectroscopy measurement sessions took place. The implanted mechanically-softening probes had stable electrochemical impedance spectra across the 16 weeks and single units were recorded out to 16 weeks. The demonstration of chronic neural recording with the mechanically-softening probe suggests that probe architecture, custom package, and general design strategy are appropriate for long-term studies in rodents.

## 1. Introduction

Intracortical neural interfaces enable both fundamental neuroscience advances and engineering strategies to restore motor, sensory, and cognitive functions to individuals who have suffered neurological injury or disease. Though electrical interfaces have dominated the field [1], recent advances in neural interfacing technologies also include single- and bi-directional chemical [2,3,4], ultrasound [5], and optical interfaces [6] for interrogating or modulating neural function. Intracortical brain-machine interfaces (BMIs) rely upon the detection of extracellular neural electrical activity in the tens-of-microvolts range using microelectrodes implanted several millimeters into the cortex. Mechanical, materials, and biological failures all contribute to the poor long-term stability and functionality of intracortical neural interfaces that continue to limit long-term, chronic studies and applications [7,8,9]. The harsh physiological environment, combined with the need to make a connection to external systems for control or recording, requires a system-level engineering design to maintain a stable interface with a microscale device requiring sensitive measurements. The probes, leads, and connectors can fail due to sudden applied forces or fatigue-related damage [9]. Electrode and insulating materials can degrade due to the harsh physiological environment, and may be accelerated by reactive oxygen species that accumulate as a result of an inflammatory tissue response to the implant [10,11,12]. Chronic inflammation also results in glial encapsulation, and further may be responsible for neuronal degradation near the implant [13,14,15]. Intracortical implant design should aim to: (1) maintain a high neuronal density at the biotic-abiotic interface, and (2) minimize chronic inflammation. Though these issues have primarily impacted intracortical probes for electrical recording, they extend to any intracortical interface and modality of interfacing, as well as to other implanted devices such as for deep brain stimulation. Therefore, engineering a reliable intracortical interface system will be impactful across a variety of applications requiring a device implanted into the cortex.

Solutions to poor long-term intracortical interface reliability largely focus on addressing the biological tissue response through geometric or materials design of neural probes [16,17,18,19,20]. Relative micromotion arising from constant, repetitive displacements in tissue due to respiration and vascular pulsations produce strain on tissue surrounding the implant due to mechanical mismatch at the implant-tissue interface for high modulus implants. The differential strain on tissue is considered to be a primary contributor to glial scar formation [21,22], which is further supported by in vitro studies reporting that components of astroglial scarring proliferate in response to mechanical strain [23] and high modulus substrates [24], while neurite outgrowth and extension is stimulated on low-modulus substrates with mechanical properties approaching brain tissue (*E*_brain_ ~ 10 kPa) [25]. Intracortical implants based on a lower modulus material reduce the differential strain on tissue during micromotion [26,27], which may also reduce the problematic neuroinflammatory response. The correlation between mechanics and the neural tissue response has led to the development of soft intracortical probes based on polymers with established microfabrication processes, such as polyimide [20,28,29], parylene [30,31,32,33], and SU-8 [34,35,36,37] polymer-based intracortical probes with much lower Young’s moduli (*E*_polymer_ ~ 2–4 GPa) than standard silicon-based devices (*E*_Si_ ~ 160 GPa) or tungsten microwire (*E*_W_ ~ 411 GPa) arrays [8,38,39], thereby alleviating implant-tissue mechanical mismatch. However, mechanically-flexible, polymer-based neural probes may buckle during implantation. In some cases, probe width or thickness of polymer-based probes may be relatively large to ensure that the critical buckling force is greater than the insertion force [37,40,41]. Several strategies have also been developed to provide enhancement of temporary stiffness, including removeable rigid shuttles to guide the probe into place and dissolvable coatings [28,33,42]. Alternatively, the effective length of the probe can be shortened to increase the critical buckling strength by partial reinforcement of the probe shank with polyethylene glycol (PEG) [43] or with the use of an insertion guide [44]. Regardless of the insertion strategy, these commercially-available polymers retain a six order-of-magnitude mechanical mismatch with brain tissue after insertion. An ideal implant for improving integration with tissue and reducing strain would have a lower modulus that more closely matches brain tissue, and can be scaled to multi-shank arrays without requiring complex removable support structures.

**Figure 1:**
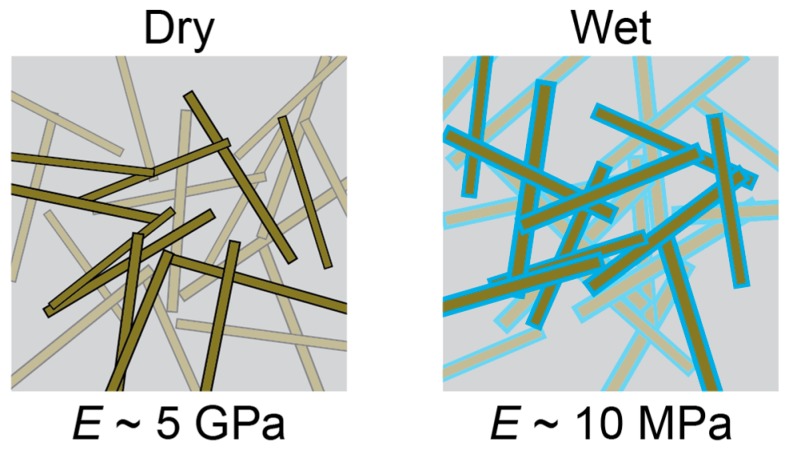
Schematic demonstrating the sea cucumber dermis-inspired PVAc-CNC softening mechanism. In the dry state (**left**), cellulose nanocrystals joined by hydrogen bonds form a reinforcing network throughout the nanocomposite. When saturated with water (**right**), the inter-nanocrystal hydrogen bonds are displaced with water molecule-nanocrystal bonds, leading to an overall reduction in storage modulus.

The biological mechanism underlying the mechanical stiffness modulation of the sea cucumber dermis inspired the development of a polymer nanocomposite with a modulus that can be controlled by temperature and degree of saturation [45,46,47]. A soft poly(vinyl acetate) (PVAc) matrix polymer with a percolated network of high-aspect-ratio cellulose nanocrystals (CNC) harvested from tunicate mantles form the nanocomposite (Figure 1). The polymer nanocomposite (PVAc-CNC) has a high Young’s modulus (*E* ~ 4–5 GPa) when dry due to the reinforcing effects of a percolating CNC network through the material. When swollen with water, the CNC network disengages, thus “turning off” the reinforcing effect. Bulk PVAc-CNC films swell 70% by weight [48], which is anisotropically distributed with a 3% increase in the lateral dimensions of water-saturated films and a 24% increase in film thickness [49]. The water-swollen matrix polymer is also plasticized, reducing the glass transition temperature (T_g_) to ~20° C. The CNC disengagement and T_g_ reduction effects combine to yield a dramatic reduction in modulus to *E* ~ 10 MPa [40,50,51]. For intracortical neural interfaces, this single material is both sufficiently rigid for needle-like insertion into tissue without buckling, while also offering softening after insertion to reduce mechanical mismatch with surrounding tissue [40]. This material reduces strain on tissue and neuroinflammation compared to standard, rigid (E ~ 160 GPa) silicon-based implants [22,26,52].

PVAc-CNC has a much wider mechanical range than other mechanically-softening polymers used for neural interface applications [18,41,53]. In its stiff state, PVAc-CNC has a modulus approximately seven times higher than the thiol-ene-based shape memory polymer [53]. In its mechanically-compliant state, PVAc-CNC has a modulus approximately four times lower than the shape memory polymer [53]. The higher stiff-state modulus for PVAc-CNC allows for a probe with a smaller cross-sectional area that will still penetrate through the pia and into the cortex without buckling. As a result of a smaller required cross-sectional area and a lower compliant-state modulus, the bending stiffness of PVAc-CNC implants can have a bending stiffness less than 5% of the bending stiffness of a shape memory polymer neural interface with the same length. However, PVAc-CNC has more extensive fabrication process limitations compared to the shape memory polymer [53,54]. Specifically, PVAc-CNC is incompatible with wet chemicals and with temperatures exceeding 100 °C [49,55]. Exposure to acids and bases will interfere with the surface properties of the cellulose nanocrystals, exposure to organic solvents will dissolve the PVAc matrix, and temperatures exceeding 100 °C will cause CNC degradation [56]. Further, PVAc-CNC is dependent upon water absorption to soften and therefore: (1) cannot serve as an insulating moisture barrier for thin-film metal traces and electrodes, and (2) cannot be completely coated with an insulating moisture barrier film.

Intracortical interfaces based upon PVAc-CNC require processes for forming a neural probe geometry and functionalizing the material with microelectrodes for recording, as well as a robust packaging system for making connection to external electronics. A complete system must consider the biological system, material properties, and forces to which the system is subjected during and after the insertion procedure. The design and method of packaging microscale neural interfaces are critical, yet often neglected, components of the implant system. For the mechanically-softening PVAc-CNC, the package must include a compatible method for making electrical connection between a rigid commercial connector and the mechanically-softening probe. The electrical interconnections between the probe and the connector must be insulated from the physiological environment and should avoid mechanical failure modalities. The connector itself must be protected to prevent mechanical breakage or removal. Finally, the entire headcap must be anchored securely to the skull for successful chronic studies. We previously reported on the fabrication and benchtop studies of an early-stage PVAc-CNC neural probe [49,51,55]. These studies demonstrated the feasibility of using PVAc-CNC as a mechanical substrate for microfabricated neural interfaces, that the thin-film metal and insulation layers do not contribute significantly to the mechanical behavior of the device, and that the device architecture remains stable through 60 days of soaking under physiological conditions [49,51,55]. Advancing PVAc-CNC neural interfaces to use in chronic studies required the refinement of the fabrication processes to include multiple microelectrodes along the shank, as well as a packaging scheme compatible with PVAc-CNC and the demands of chronic implantation. Here, we report on the progress we have made toward advancing PVAc-CNC neural interfaces to chronic implant studies.

## 2. Materials and Methods 

### 2.1. Design and Overview

Our goal was to produce a planar microelectrode array with up to 8 recording sites on the PVAc-CNC polymer nanocomposite structural material. Planar microelectrode arrays allow for simultaneously measuring from multiple depths within the cortex [57]. The PVAc-CNC neural probe has a five-layer architecture comprising a PVAc-CNC structural substrate layer, a Parylene C barrier layer, Au electrodes and interconnections with a Ti adhesion layer, and a Parylene C capping layer. Additionally, probe length, width, and thickness must be chosen such that the critical buckling force, as determined by Euler’s buckling formula, is greater than the force required to insert the probe [40]. The probe must be able to withstand an insertion force of 10 mN, based upon a typical insertion force of 5 mN [40] and a safety factor of 2. For a 40 µm-thick PVAc-CNC probe with a length of 3 mm, the probe width must be at least 140 µm.

### 2.2. Materials

#### 2.2.1. PVAc-CNC

The polyvinyl acetate-cellulose nanocrystal polymer nanocomposite serves as the structural material for the neural probe. The methods for synthesizing PVAc-CNC have been described in detail elsewhere [45,46,48,58]. Briefly, poly(vinyl acetate) was dissolved in dimethylformamide (DMF). Cellulose nanocrystals were dispersed in DMF in a second beaker. The two solutions were mixed, then cast into a Teflon dish before drying under vacuum at 65 °C for 7 days [48]. The dry PVAc-CNC films were then pressed to a thickness of 30–60 µm at a temperature of 90 °C and a pressure of 3000 psi [59]. 

#### 2.2.2. Parylene C

Parylene C serves as an insulating moisture barrier for the interconnection traces between the recording sites and connection contacts. Parylene is an FDA Class VI material and is a good moisture barrier with a 24-h water absorption of 0.06% and 0.14 g-mil/100 in^2^ for 24 h at 37C, 90%RH moisture vapor transmission [60]. The Parylene C used in this application was vapor deposited with a Specialty Coating Systems Labcoter^®^ 2 Parylene Deposition System (Specialty Coating Systems, Inc., Indianapolis, IN, USA).

#### 2.2.3. Au/Ti

Sputter-deposited, thin-film Au was chosen for the microelectrode recording sites, connector contacts, and interconnecting traces due to its biocompatibility, inertness, and low residual stress. 

### 2.3. Fabrication

**Figure 2:**
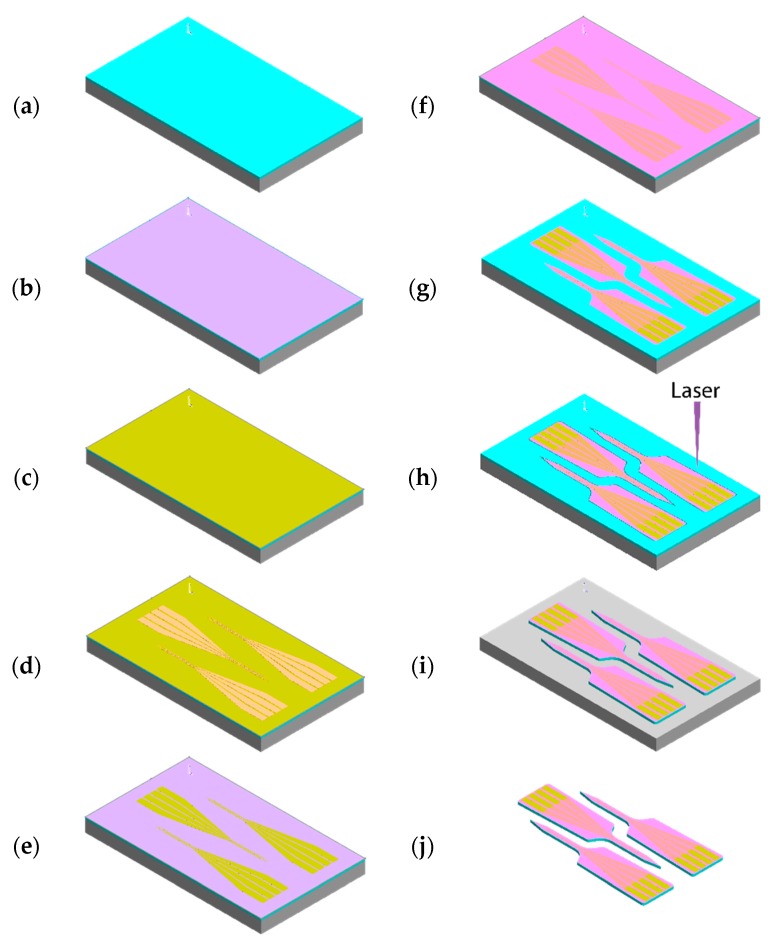
Microfabrication process for PVAc-CNC intracortical probes. (**a**) PVAc-CNC film mounted to bare Si probe; (**b**) Parylene C deposition; (**c**) Ti/Au deposition; (**d**) Photoresist spin-coating and patterning; (**e**) Wet etching of Ti/Au films; (**f**) Second Parylene C deposition; (**g**) Parylene C patterned using oxygen plasma; (**h**) Laser micromachining the PVAc-CNC substrate; (**i**) Remove excess PVAc-CNC in field region; (**j**) Release probes.

The PVAc-CNC neural probe fabrication process averts PVAc-CNC exposure to wet chemicals or temperatures exceeding 100 °C. Exposure to acids and bases will interfere with the surface properties of the cellulose nanocrystals, exposure to organic solvents will dissolve the PVAc, and temperatures exceeding 100 °C will cause CNC degradation [56]. The microfabrication steps are illustrated in Figure 2a–j. First, a freestanding PVAc-CNC film was prepared by solution-casting and melt-pressing [48]. A silicon wafer provided a rigid support for the PVAc-CNC film during the fabrication process. The PVAc-CNC film was adhered to a silicon wafer by heating the assembly to 75 °C on a hotplate and pressing the film onto the silicon wafer (Figure 2a). It was important to avoid air bubbles between the wafer and the PVAc-CNC film. Next, a 2 µm-thick Parylene C barrier layer was vapor-deposited onto the PVAc-CNC film and silicon wafer (Figure 2b). This layer provided a moisture barrier necessary for protecting the PVAc-CNC film during wet chemical processing steps. Additionally, this layer was required to insulate the thin-film Ti/Au features from the electrolytic fluid absorbed by PVAc-CNC in vivo [49,55]. Next, a 20 nm-thick Ti adhesion layer and a 250 nm-thick Au conductive layer were sputter-deposited on the parylene film (Figure 2c). The Ti/Au films were patterned by photolithography using an iodine-based Au etchant (Gold Etch Type TFA, Transene Company, Inc., Danvers, MA, USA) and a buffered oxide etchant (Buffered Oxide Etchant 7:1 with Surfactant, Transene Company, Inc., Danvers, MA, USA), followed by removal of the photoresist with acetone and isopropanol (Figure 2d,e). A second 2 µm-thick parylene layer was then vapor-deposited to provide a capping layer to insulate conductive interconnect traces (Figure 2f). Openings in the Parylene C at the recording sites and connector contacts, as well as the outer geometry of the Parylene C layers were etched using reactive ion etching (RIE) with O_2_ and CF_4_ through a photoresist mask (Figure 2g). The outer probe geometry was then defined by laser-micromachining with a picosecond laser (Oxford Lasers, Didcot, UK) (Figure 2h). The excess material between probes was peeled from the handle wafer (Figure 2i). Finally, the completed probes were removed from the wafer with the aid of a razor blade (Figure 2j).

### 2.4. Packaging

**Figure 3:**
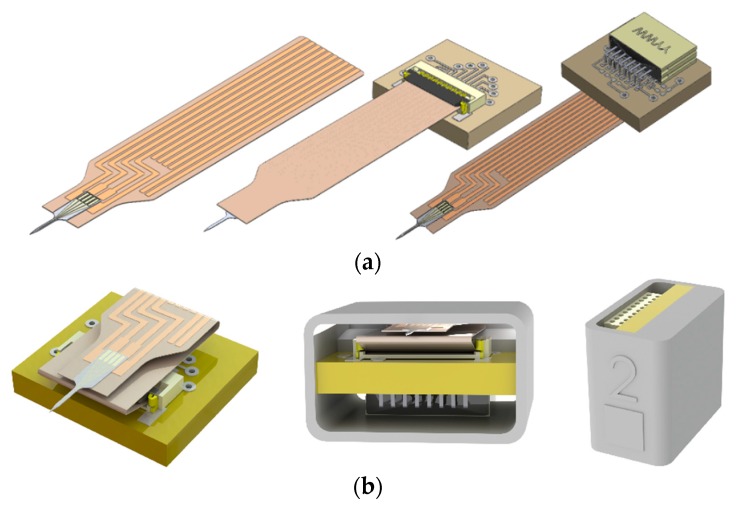
Packaging scheme for PVAc-CNC intracortical probes. (**a**) The probes are attached to a polyimide-based ribbon cable with patterned Cu traces. The ribbon cable is designed for insertion into a flexible circuit connector mounted on a printed circuit board with an Omnetics connector for interfacing with external electronics; (**b**) The ribbon cable/PCB/connector assembly is inserted into a custom-designed, 3D-printed housing to protect the components.

The packaging scheme was designed to be modular such that the components could be tested at various levels of assembly and could be modified for compatibility with alternate applications. Probes were directly attached to polyimide-based ribbon cables (Pyralux, DuPont, Wilmington, DE, USA) designed to interface with a Hirose FH-19 Flexible circuit board connector (Hirose Electric Group, Shinagawa, Tokyo, Japan). Both the flexible circuit connector and an Omnetics Nano Strip connector (Omnetics Connector Corporation, Minneapolis, MN, USA) were mounted to a printed circuit board (Figure 3a), providing a means to connect to external electronics for neural recording and for impedance measurements. A 3D-printed housing was custom-designed and fabricated to hold the PCB, connectors, and ribbon cable (Figure 3b). 

The ribbon cables were fabricated by etching the Pyralux copper cladding with a sodium persulfate solution through a laser-printed toner etch mask. The toner was then removed from the surface of the copper using acetone. The copper traces were insulated with an acrylic spray through a shadow mask, then the ribbon cable was cut out by laser micromachining. Probes were attached to the flexible ribbon cable using a cyanoacrylic adhesive. Electrical connection between the copper ribbon cable pads and the gold probe pads was made with conductive epoxy (MG Chemicals 8331S, MC Chemicals, Surrey, British Columbia, Canada). To insulate the electrical connections, a 2-part epoxy was applied using a needle to the exposed conductive epoxy and contact pads on both the probe and ribbon cable. The ribbon cables were inserted into the flexible circuit connector on the PCB, then the assembly was pushed into the 3D-printed housing. The PCB slides into one section of the housing, while a separate section holds the ribbon cable to ensure that the probe shank was normal to the housing. The components were secured with insulating epoxy.

### 2.5. Benchtop Impedance Measurements

Fully-packaged PVAc-CNC probes were immersed in phosphate buffered saline in a heated water bath at 37 °C, then the impedance spectra of recording sites were measured over 48 days. An EZStat Pro potentiostat (NuVant Systems Inc., Crown Point, IN, USA) was used to measure the impedance versus a platinum reference wire between 10 Hz and 10 kHz, with 25 points per decade. The impedance magnitude at a frequency of 1 kHz was used to compare frequency over time.

### 2.6. Chronic In Vivo Experiments

#### 2.6.1. Surgical Procedure

Three male Sprague-Dawley rats (225–250 g) were implanted with a single PVAc-CNC neural probe in the primary motor cortex, which was then left in place for 16 weeks. The PVAc-CNC probes were sterilized using ethylene oxide. All procedures and animal care practices were approved by and performed in accordance with the Case Western Reserve University Institutional Animal Care and Use Committee. The surgical procedures followed standard protocols [52,61,62]. Briefly, the rats were initially anesthetized by a mixture of ketamine (80 mg/kg) and xylazine (10 mg/kg) administered intraperitoneally (IP). After preparing the animal’s head by shaving and cleaning, a one-inch incision was made down the midline; then the surrounding tissue was retracted to expose the skull. An opening approximately 3 mm in diameter was drilled into the skull in the left hemisphere approximately 3 mm lateral to the midline and 2 mm anterior to bregma. The dura was deflected using a dura pick to expose the pia. Three stainless steel screws (#2-56) were implanted in the skull, and the ground and reference wires were attached to the base of two of the screws, then secured in place with silver print. The probe was brought within 2 mm of the brain surface for positioning while ensuring that the shank remained dry. Once in place, the probe was rapidly lowered using a manual micromanipulator at a rate of approximately 0.5 mm∙s^−1^ to a final depth of approximately 2 mm. After the PVAc-CNC probe and housing were set in place, silicone elastomer (Kwik-Sil, World Precision Instruments) was applied to seal the craniotomy. The connector housing was secured in place with dental acrylic anchored by the screws. Finally, the skin on the scalp was closed around the housing with 1–2 sutures.

#### 2.6.2. Neural Recording and EIS Measurements

Eleven neural recording sessions took place during the 16-week implant duration. Each recording session lasted for approximately 10 min and involved cleaning and drying the housing and Omnetics connector, connecting a pre-amplifier and cable, then allowing the rat to freely move within a clean cage. Neural potentials were recorded with a 16-channel Tucker David Technologies (TDT) Pentusa Z5 system (Tucker-Davis Technologies, Alachua, FL, USA), using a sampling rate of 24.4 kHz. EIS measurements were made with the rat under isoflurane anesthesia using an amplitude of 10 mV between 1 Hz and 10 kHz with 10 frequencies per decade. 

#### 2.6.3. Neural Recording Data Analysis

Data from each trial was processed by a MATLAB (R2013, MathWorks, Natick, MA, USA) analysis program based on code from the Kipke Lab [63,64]. Signals were separated into local field potential (LFP) (0.1–140 Hz) and neural spike (300–5000 Hz) components in MATLAB. For spike analysis, a negative threshold at 3.5 times the standard deviation of the signal was set to identify candidate samples. Any sample crossing the threshold was considered for further processing. The samples were considered within a 2.4 ms window, and the minimum potential was chosen to be the center of the spike snippet window at 1.2 ms. A principal component analysis (PCA) was performed on a voltage amplitude matrix (*N*_spikes_ × 100 timepoints) corresponding to the collection of spike snippet windows to cluster the spikes. Neural units were identified by choosing the clusters with more than 20 spikes in a cluster. A mean spike waveform was created from the spikes in the cluster, and the peak-to-peak voltage of the mean waveform determined the peak-to-peak signal voltage. The peak-to-peak noise voltage of each channel and block was determined by first removing the spike snippets windows from the signal and determining the standard deviation of the remaining signal. The peak-to-peak noise voltage was then defined as 3 times the standard deviation of the remaining noise signal. 

## 3. Results and Discussion

### 3.1. Device Fabrication

**Figure 4:**
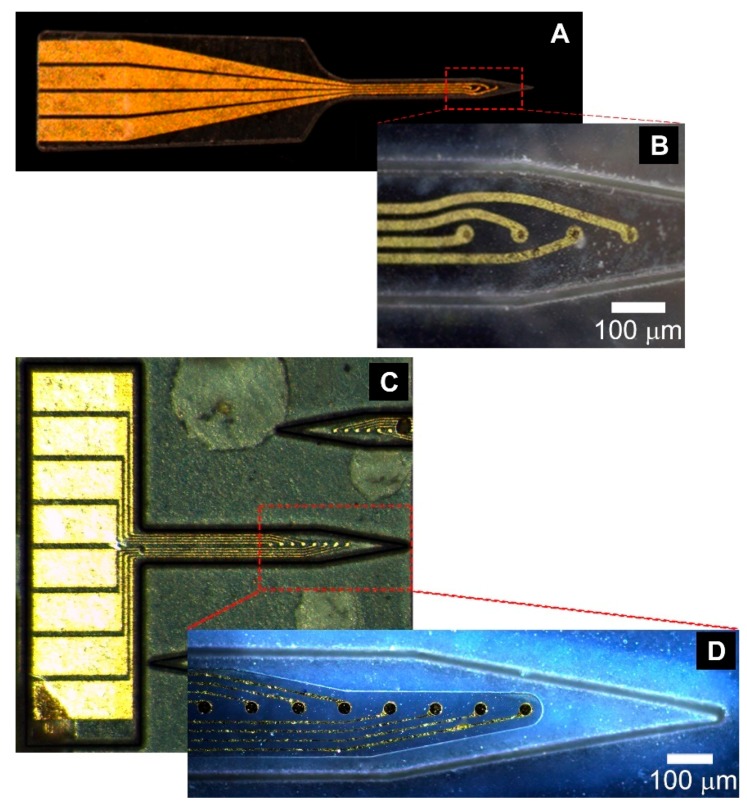
PVAc-CNC neural probes: (**A**) 4-channel probe overview; (**B**) Close-up of 15 µm-diameter Au microelectrode sites on 4-electrode probe; (**C**) 8-channel probe overview; (**D**) Close-up of 30 µm-diameter Au microelectrode sites on 8-electrode probe.

Multi-electrode arrays with between 4 and 8 individually-addressable microelectrode recording sites were fabricated on the PVAc-CNC polymer nanocomposite, as shown in Figure 4. Thin-film metal feature sizes down to 7 µm were resolved on the Parylene C-coated PVAc-CNC surface. The solution-cast and compressed PVAc-CNC films have surface height variations of up to 4 µm over a 100 µm lateral distance, yielding a much larger surface roughness than a standard silicon wafer with sub-nanometer surface roughness. The minimum resolvable feature size is therefore limited by the roughness and uniformity of the PVAc-CNC surface, and can be decreased further by refining PVAc-CNC film manufacturing processes. An important aspect of our refined fabrication process is the use of a picosecond UV micromachining laser with alignment capability to pattern the PVAc-CNC probe outer geometry after the photolithography steps, instead of as one of the first fabrication steps. This allowed for photolithography steps on a much more planar surface, thus improving yield and minimum feature size. In the 4-electrode design shown in Figure 4A,B, the parylene capping layer covers the entire front-side of the probe. To maximize the benefits of the PVAc-CNC material properties, the Parylene C footprint was minimized in the 8-electrode design shown in Figure 4C,D. This change in geometry was made possible with improved precision in photolithography, etching, and PVAc-CNC patterning. These improvements also facilitated a reduction in interconnect trace width from 15 µm to 7 µm without sacrificing yield. This microfabrication process is scalable and can be used for multiple shanks or to increase the number of recording sites per shank.

Device yield was largely dependent upon the elimination of air from between the PVAc-CNC film and the underlying silicon handle wafer. Unlike spin-cast or vapor-deposited polymers, PVAc-CNC begins as a free-standing film. Air trapped between the wafer and PVAc-CNC film can expand during microfabrication steps that occur under vacuum, particularly during sputter deposition when the nanocomposite softens with an increase in temperature. Additionally, the in-line design of the 4-channel design had fewer mechanical failures during release from the wafer than the wide connector contact pad layout with the sharp transition to the shank featured in the 8-contact design.

### 3.2. Probe Packaging

**Figure 5:**
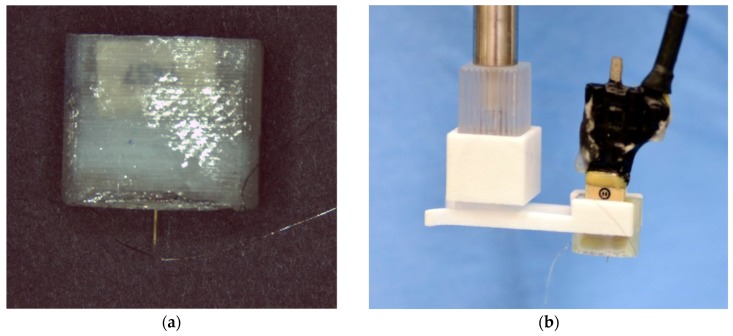

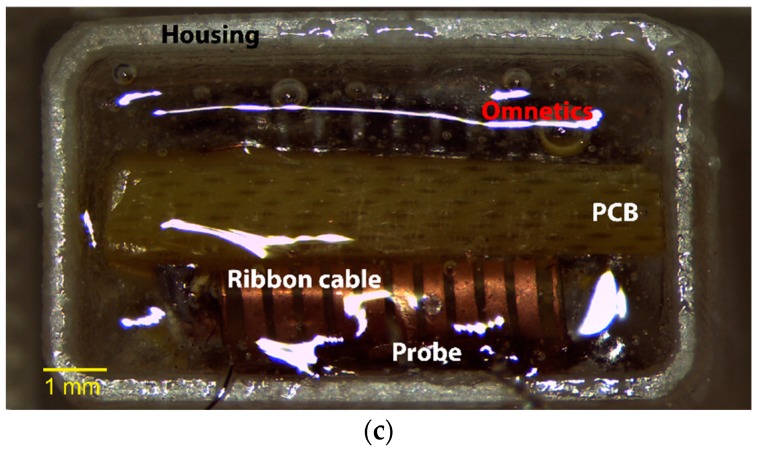
Packaged PVAc-CNC neural probes: (**a**) Front-view of probe in connector housing; (**b**) Assembly held with a custom clip for insertion; (**c**) Underside view of probe in connector housing, which shows the ribbon cable folded into the connector housing.

We considered materials and process compatibility, modularity, and robustness when designing and developing a package for the PVAc-CNC probes. In our packaging scheme, the probe, reference, and ground wires were attached to a polyimide-based Pyralux ribbon cable with copper traces running from the probe to an end designed to interface with a flexible circuit connector. Electrical connection between the Au contact pads on the probe and the Cu contact pads on the ribbon cable was made with Ag-based conductive epoxy. The contact resistance between the Au contact pads on the probe and the Cu contact pads on the ribbon cable was less than 5 Ω, which is negligible compared to the overall trace resistance and electrode-electrolyte interface impedance. The ribbon cable provides a modular approach to making electrical connection to the probe, thus allowing for testing before assembling the complete package. Further, electrical connection via a ribbon cable lends itself to design flexibility, as the ribbon cable length can be increased to reduce tethering forces, and the configuration can be modified to enable more bending and stretching without putting undue stress on the ribbon cable traces. In our in vivo studies, the ribbon cable was folded and inserted into the 3D printed housing (Figure 3 and Figure 5). The ribbon cable connected to a printed circuit board via a flexible circuit connector (Hirose FH-12-10SH). The neural recording system was then connected via cable to an Omnetics Nanostrip connector also mounted on the PCB. The completed assembly is shown in Figure 5. The housing made it possible to grip the housing for insertion purposes and protect the circuit board and all connections while implanted. The package can be easily scaled up to accommodate more recording contacts.

The labor-intensive process required to package the PVAc-CNC probes presents several risks for failure of a mechanically-brittle probe. To reduce these risks and improve yield of packaged probes, PVAc-CNC probes are packaged while the shanks are slightly moist and are therefore less brittle. Future designs will include a monolithically-integrated ribbon cable that will reduce the level of skill required to make a connection between the PVAc-CNC and connectors to external electronics.

### 3.3. Benchtop Characterization

**Figure 6:**
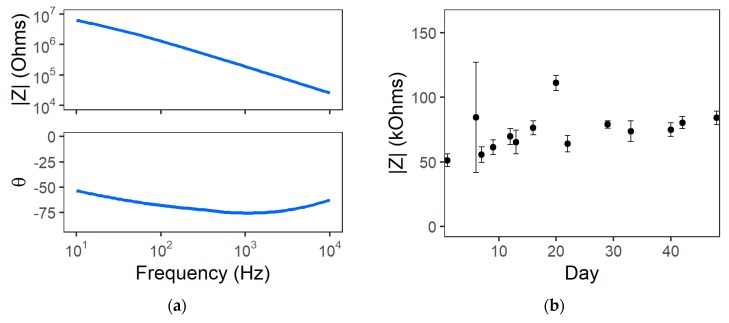
Representative results from PVAc-CNC soak testing with 50 µm-diameter microelectrodes: (**a**) Impedance spectra at 1 h after immersion in PBS; (**b**) Impedance magnitude at a 1 kHz frequency over time.

EIS results for the 50 µm-diameter PVAc-CNC microelectrode sites in PBS indicated impedance magnitude values between 55.1 and 190.7 kΩ at a frequency of 1 kHz. EIS results from a typical recording site are shown in Figure 6a. The average impedance magnitude at a frequency of 1 kHz of six channels across two devices measured over 48 days is shown in Figure 6b. The electrode-electrolyte interface properties are typical for 50 µm-diameter Au microelectrodes and remained relatively stable throughout the soak test. These results indicated that the microelectrodes remained intact and the Parylene C served as a moisture barrier for the soak test duration. Based on this data, we determined that these probes were sufficiently robust for preliminary in vivo investigations for chronic recording and electrochemical impedance spectroscopy measurements.

### 3.4. Chronic Implant Experiments

#### 3.4.1. Surgery/Insertion

Using the custom, 3D-printed clip to hold the probe housing, the probes were inserted into the cortex cleanly and securely. The insertion needed to be performed within a few seconds due to the rapid softening of the PVAc-CNC material [40,49,50]. The headcaps remained firmly in place for the 16-week duration of the experiment. 

PVAc-CNC swelling is primarily a concern only during the insertion process because swelling corresponds to a reduction in material stiffness. Further, damage during implantation can be minimized by completing the insertion process before the probe is able to swell appreciably. Though an increase in implant size has generally correlated with an increase in glial scarring and a decrease local neuronal density [65,66], a decrease in mechanical modulus [26,52,67] and material density [68] of the structural material can improve the tissue response and neuronal density. PVAc-CNC swelling is anisotropic, favoring the through-thickness dimension by 8-fold. The minimal swelling across the film prevents probe curling or bending, even though one surface of the film is constrained by the parylene films, electrodes, and interconnects. Though additional study is required to understand the effects of PVAc-CNC swelling on recording quality, we speculate that through-thickness swelling may have a positive effect on neural recording quality by reducing the distance between recording electrode sites and active neurons after deployment.

#### 3.4.2. Electrochemical Impedance Spectroscopy

**Figure 7:**
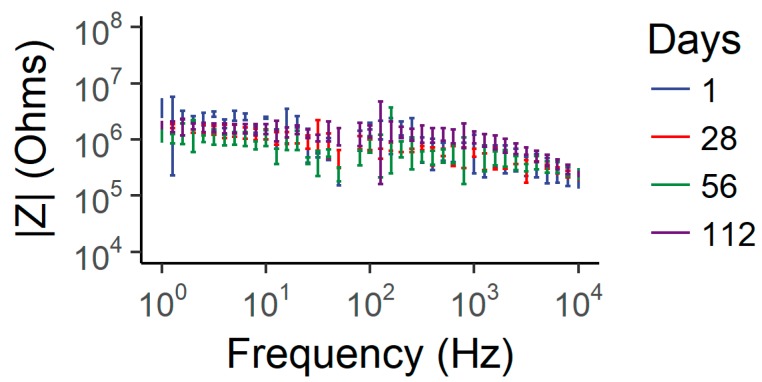
Comparison of impedance magnitude spectra from EIS over the 16-week implant period.

EIS results, averaged across functional channels, measured across the duration of the implant time period, are shown in Figure 7. At 1 kHz, the impedance magnitude initially ranges from 0.32 to 1.28 MΩ, which increases to a range of 0.82–1.31 MΩ on the final day. The smaller area of the implanted microelectrodes sites (15 µm-diameter) compared to the microelectrode sites on the soak-tested devices (50 µm-diameter) (Figure 6) resulted in a higher electrochemical impedance. These valued scaled as expected with 1/r^2^ [69]. Further, these are typical impedance values for gold microelectrodes of a similar area [70]. Overall, there is no clear trend in impedance magnitude over the 16 weeks, and the spectra are quite stable across the duration. This stability indicates that the electrode contacts, insulating barrier and capping layers, and the surrounding tissue properties remained quite stable for the duration of the implant duration.

#### 3.4.3. Chronic Neural Recording

**Figure 8:**
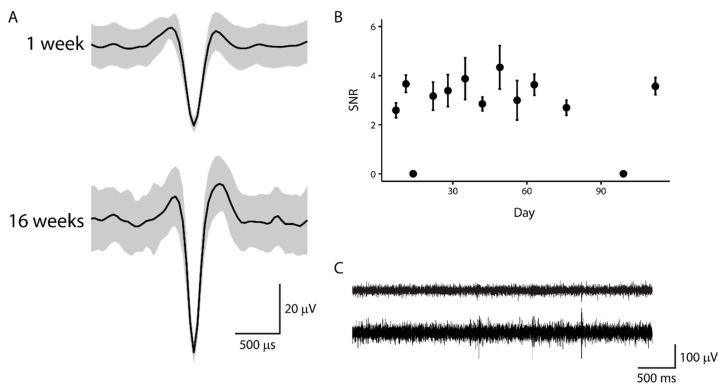
Demonstration of neural recording with PVAc-CNC neural probes. (**A**) Average waveform from 50 isolated and clustered spike snippets at the1-week timepoint (top) and 16-week timepoint (bottom); (**B**) Mean SNR +/− s.e. for isolated units detected for each recording session during the 16-week implant period; (**C**) High-pass (>300 Hz) filtered traces for two adjacent microelectrodes at the 16-week timepoint.

Neural activity with a signal-to-noise ratio between 2.6 and 4.3 was recorded using the PVAc-CNC neural probes during the 16-week implant duration (Figure 8B). Average unit waveforms from pile-plots of 50 isolated spike snippets recorded at 1-week (Figure 8A, top) and 16-week (Figure 8A, bottom) timepoints indicate that the PVAc-CNC probes are sufficiently robust in terms of probe architecture and packaging to be able to record isolated units at a 16-week timepoint. The mean signal-to-noise ratio (SNR) for isolated units from each recording session with the same probe is shown in Figure 8B. The SNR was relatively stable, especially beyond the 30-day timepoint. There were two recording sessions during which no action potentials were recorded. With the exception of these two recording sessions, isolated units were recorded on at least one channel for each recording session. For some sessions, units were recorded on two or three channels. High-pass filtered (>300 Hz) voltage traces recorded from adjacent channels at the 16-week timepoint are shown in Figure 8C. Each trace contains unique features and spikes, indicating that crosstalk between traces is minimal, even at the 16-week timepoint. These results are the first demonstration of neural recording using the PVAc-CNC material, and therefore offers encouraging results for the chronic functional use of the mechanically-softening polymer nanocomposite.

## 4. Conclusions

We developed a neural probe system based on a mechanically-adaptive polymer nanocomposite using a microfabrication process flow designed for compatibility with the chemical and thermal sensitivities of PVAc-CNC. Importantly, the polymer nanocomposite was designed specifically for biocompatibility and biological integration as an implant material, which contrasts strongly with the focus on processability for silicon- and mechanically-static polymer-based neural implants. PVAc-CNC is a sufficiently rigid material in its dry state to penetrate the cortex before dramatically softening and reducing mechanical mismatch at the interface. To overcome the incompatibility of PVAc-CNC with standard microfabrication processes, the process flow used to develop PVAc-CNC neural probes was reliant upon laser micromachining and the use of a conformal Parylene C coating that protects PVAc-CNC from exposure to wet chemicals during processing and insulates thin-film traces from electrolytic fluid absorbed by PVAc-CNC while implanted. A custom, robust package was also designed to interface the microscale polymer nanocomposite-based probes with external electronics and maintain the ability to connect to external electronics for at least 16 weeks. For the first time, we have demonstrated that the PVAc-CNC probes remain electrically functional and stable for an extended duration and are capable of recording electrical neural activity for at least 16 weeks.
